# Predicting the fundamental thermal niche of ectotherms

**DOI:** 10.1002/ecy.4289

**Published:** 2024-04-05

**Authors:** Margaret W. Simon, Priyanga Amarasekare

**Affiliations:** Department of Ecology and Evolutionary Biology, University of California, Los Angeles, California, Los Angeles, USA

**Keywords:** climate envelope, conditions for population viability, delay differential equation population model, density-independent abundance, temperature response of abundance, temperature response of life-history traits

## Abstract

Climate warming is predicted to increase mean temperatures and thermal extremes on a global scale. Because their body temperature depends on the environmental temperature, ectotherms bear the full brunt of climate warming. Predicting the impact of climate warming on ectotherm diversity and distributions requires a framework that can translate temperature effects on ectotherm life-history traits into population- and community-level outcomes. Here we present a mechanistic theoretical framework that can predict the fundamental thermal niche and climate envelope of ectotherm species based on how temperature affects the underlying life-history traits. The advantage of this framework is twofold. First, it can translate temperature effects on the phenotypic traits of individual organisms to population-level patterns observed in nature. Second, it can predict thermal niches and climate envelopes based solely on trait response data and, hence, completely independently of any population-level information. We find that the temperature at which the intrinsic growth rate is maximized exceeds the temperature at which abundance is maximized under density-dependent growth. As a result, the temperature at which a species will increase the fastest when rare is lower than the temperature at which it will recover from a perturbation the fastest when abundant. We test model predictions using data from a naturalized–invasive interaction to identify the temperatures at which the invasive can most easily invade the naturalized’s habitat and the naturalized is most likely to resist the invasive. The framework is sufficiently mechanistic to yield reliable predictions for individual species and sufficiently broad to apply across a range of ectothermic taxa. This ability to predict the thermal niche before a species encounters a new thermal environment is essential to mitigating some of the major effects of climate change on ectotherm populations around the globe.

## INTRODUCTION

Climate change is predicted to increase global temperatures and generate more extreme temperature fluctuations (IPCC, 2018; Sherwood et al., 2020). There is increasing evidence that warming facilitates the invasion success of exotic species (Dukes & Mooney, 1999; Sorte et al., 2013; Stachowicz et al., 2002), predisposes native biota to extinction (Parmesan, 2006; Pereira et al., 2010; Root et al., 2003; Walther et al., 2002, 2009), and increases outbreaks of pests and pathogens (Bebber et al., 2013; Harvey et al., 2020; Lehmann et al., 2020). Ameliorating the joint effects of climate warming and invasive species is one of the major environmental challenges of this century (Hellmann et al., 2008; Sala et al., 2000).

**FIGURE 1:**
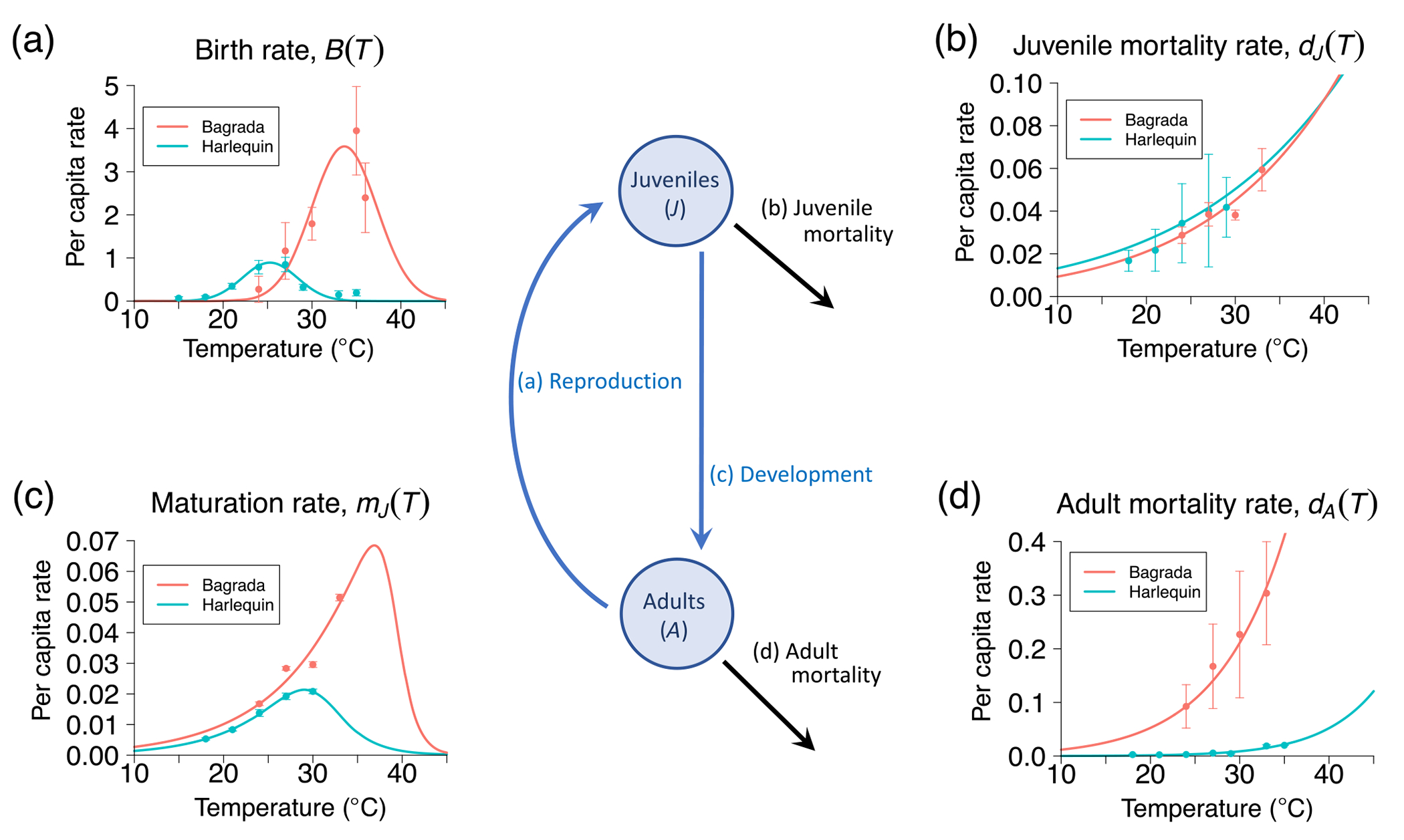
Conceptual diagram of typical ectotherm life cycle and temperature responses of life-history traits for bagrada (red curves) and harlequin bug (blue curves) in absence of density dependence (representing Equations 1–4). Juveniles develop into adults over a temperature-dependent duration $(T)\left(\tau (T)=\frac{1}{m_{J}(T)}\right)$, and adults produce new juveniles at birth rate B(T). Mortality, $d_{J}(T),d_{A}(T)$, can occur at either stage. Panels (a)–(d) depict, respectively, the temperature responses of birth, juvenile mortality, maturation, and adult mortality rates in units per day. Solid circles with error bars depict observed trait responses, and curves depict temperature responses predicted using parameterized response functions (Equations 10–12, Table 1). Density dependence can alter these rates in a fashion that is monotonically increasing or unimodal in response to temperature (Appendix S1: Figure S1).

The vast majority of biodiversity on the planet consists of ectotherms, species that cannot physiologically regulate their body temperature (e.g., microbes, invertebrates, fish, amphibians and reptiles). Nearly all pests and pathogens are also ectotherms. Temperature variation directly affects the physiology, behavior, and population dynamics of such species. Ectotherm life-history traits (e.g., fecundity, development, survivorship) exhibit responses to temperature variation that are plastic, that is, when the temperature changes the response changes accordingly (Figure 1a–d). Population- and community-level responses to climate change—shifting of species’ ranges, competitive displacement of native species by invasive species, emergence of new pathogens—ultimately arise from these plastic responses that occur at the level of individual organisms. For example, thermal plasticity in ectotherm development rates allow for population-level phenological changes such as earlier or later emergence (CaraDonna et al., 2014; Forrest, 2016; Miller-Rushing et al., 2010; Post et al., 2018; Scranton & Amarasekare, 2017; Vitasse et al., 2022). It is these individual-level responses on which we need to focus if we are to predict the impact of warming on biodiversity and invasive species. The challenge is to determine how these individual-level responses translate into population- and community-level patterns.

The metric that makes this translation possible is the fundamental thermal niche, the range of temperatures over which a species can maintain a positive intrinsic growth rate. The typical approach is to use observed abundances across space to quantify a species’ thermal niche, for example, by using maximum entropy approaches to relate species observations and the temperatures for which such observations occurred (Buckley et al., 2010). However, this has the drawback that the observed pattern is the result of both abiotic and biotic factors and, hence, represents a species’ realized thermal niche, not the fundamental niche. A species might be abundant at a particular location simply because it is released from competitors or natural enemies and not because the temperature is optimal for population growth. Making reliable predictions of warming effects on population viability and species’ distributions requires an alternative approach.

Here we present a mechanistic theoretical framework that allows us to predict a species’ fundamental thermal niche based solely on how temperature affects the species’ life-history traits, completely independently of any population-level information. We build on recent theory that predicts temperature effects on ectotherm fitness and population dynamics (Amarasekare & Coutinho, 2013, 2014; Amarasekare & Savage, 2012). We take advantage of large-scale data analyses showing that the qualitative nature (e.g., left-skewed, Gaussian) of trait responses to temperature (i.e., thermal reaction norms) is conserved across ectotherm taxa and that their parameter values are thermodynamically constrained to fall within a narrow range (Amarasekare & Savage, 2012; Brown et al., 2004; Dell et al., 2011; Englund et al., 2011; Gillooly et al., 2001, 2002; Savage et al., 2004). This allows us to build a general framework that applies broadly across ectotherm taxa, habitats, and latitudes.

Our framework incorporates temperature responses of ectotherm life-history traits into a stage-structured population model that realistically captures the complex life cycles of multicellular ectotherms (Figure 1). We use the model to derive a relationship between a species’ temperature-dependent intrinsic growth rate and its abundance in the absence of population regulation. We also predict long-term abundance when population regulation is itself temperature-dependent. Since the trait-based framework allows us to calculate the intrinsic growth rate, long-term abundance, and the recovery time (time for a population to return to steady state following a disturbance in population dynamics) at any biologically relevant temperature, we can determine the temperature for which a species will (1) increase when rare at the fastest rate, (2) exhibit the greatest long-term abundance under resource limitation, and (3) recover from a perturbation to the steady state most quickly. Together, these metrics enhance predictive understanding of the response of native species under warming as well as potential outcomes (establishment or extinction) of species introductions into novel thermal environments. We apply these concepts to investigate the interaction between a naturalized insect species in the California coastal sage scrub and an exotic species that has recently invaded this habitat.

## CONCEPTUAL FRAMEWORK

In the absence of density-dependent (DD) factors, a population will grow at its intrinsic growth rate, r. When r>0, the population can increase from initially small numbers. We can define the fundamental niche as the range of abiotic conditions over which a species can maintain a positive intrinsic growth rate (Holt, 2009).

This characterization of the fundamental niche is straightforward in principle but difficult to estimate in practice. The prevailing approach is to measure abundance data in the field and calculate the intrinsic growth rate as the average rate of change in population size between consecutive sampling periods having similar abiotic conditions. However, abundance observed in the field is the outcome of both abiotic conditions and biotic interactions. A species may be absent from a given location simply because it has been excluded by a competitor, overexploited by a predator, or has not yet arrived there due to dispersal limitation. Thus, calculation of the intrinsic growth rate in the field using abundance data could unknowingly incorporate, for example, ongoing biotic interactions that impose DD feedbacks on the per-capita growth rate. We need a way to relate the intrinsic growth rate to abundance so that we can predict the range of temperatures over which a species attains nonzero abundance in the absence of competitors, natural enemies, and dispersal limitation.

We make this connection by developing a population model that incorporates the (1) stage-structured life cycle characteristic of all multicellular ectotherms and (2) mechanistic descriptions of life-history trait responses to temperature (Figure 1). We consider an ectotherm species whose life cycle consists of a nonreproductive juvenile stage (e.g., eggs, larvae, nymphs) and a reproductive adult stage. The juvenile stage transitions to the adult stage through the process of development, which involves both maturation and mortality. Maturation from juvenile to adult introduces a time delay between when the current adult population reproduces and when the resulting offspring become adults. Since some juveniles die during maturation, not all reproductive events lead to a new adult. A biologically realistic model for ectotherm population dynamics requires incorporating both the developmental delay and mortality during maturation. We do this using delay differential equations (DDEs), which provide the analytical means of incorporating delays and their temperature dependence into a stage-structured population model. We use the DDE framework to derive necessary and sufficient conditions for population viability.

### Necessary condition for viability

The necessary condition for viability is that a species be able to increase from initially small numbers, that is, its intrinsic growth rate should be positive (Chesson, 2000). We use a stage-structured model with density-independent (DI) population dynamics to derive the temperature response of the intrinsic growth rate:

(1) $$\frac{dJ(t)}{dt}=b[T(t)]A(t)-M_{J}(t)-d_{J}[T(t)]J(t)\;\frac{dA(t)}{dt}=M_{J}(t)-d_{A}[T(t)]A(t),$$

where J and A depict juvenile and adult abundances, and the functions b[T(t)] and $d_{X}[T(t)]$ describe the temperature-dependent birth and mortality rates (X=J,A). Note that the temperature upon which these functions depend, T(t), is itself a function of time. The function $M_{J}(t)$ depicts the total maturation rate of juveniles into adults and is given by

(2) $$M_{J}(t)=b[T(t-\tau (t))]A(t-\tau (t))\frac{m_{J}[T(t)]}{m_{J}[T(t-\tau (t))]}S_{J}(t).$$

Juveniles maturing into adults at any given time t are those born at time t-τ(t) via adult reproduction at that time, where τ(t) is the length of the maturation period. The birth rate at time t-τ(t) is given by b[T(t-τ(t))]A(t-τ(t)). Not all births are successful, however, since some juveniles die during maturation at the DI rate $d_{J}[T(t)]$. Hence, the fraction of individuals surviving through the juvenile stage is $S_{J}(t)=e^{-d_{J}[T(t)]\tau (t)}$. The ratio $\frac{m_{J}[T(t)]}{m_{J}[T(t-\tau (t))]}$ depicts the temperature dependence of the per-capita maturation rate $m_{J}[T(t)]$ (see details below).

When temperature varies over time (e.g., due to diurnal and/or seasonal variation), all life-history traits become functions of time. This time dependence necessitates two additional equations to describe through-stage survivorship $S_{J}(t)$ and developmental delay τ(t) (Murdoch et al., 2003; Nisbet, 1997; Nisbet & Gurney, 1983).

When the developmental delay varies over time, we can assign each juvenile a maturation index $M_{\text{index}}$ to describe its progression through the juvenile stage. Maturation to the adult stage occurs when $M_{\text{index}}=1$ (Nisbet, 1997; Nisbet & Gurney, 1983). If $m_{J}(t)$ is the per-capita maturation rate, which varies with time in response to temperature variation, and τ(t) is the time spent in the juvenile stage by an individual maturing at time t, then $\int_{t-\tau (t)}^{t}\;m_{J}[T(t)]dx=1$. By differentiating the integral and rearranging terms (Nisbet, 1997; Nisbet & Gurney, 1983), we obtain the following DDE for the developmental delay:

(3) $$\frac{d\tau (t)}{dt}=1-\frac{m_{J}[T(t)]}{m_{J}[T(t-\tau (t))]}.$$

The ratio $\frac{m_{J}[T(t)]}{m_{J}T(t-\tau (t)]}$ determines how temperature affects maturation. If temperature increases over the juvenile stage duration, the ratio exceeds one, stage duration is shorter, and more individuals survive through the stage; if temperature decreases over the stage duration, the ratio is less than one, stage duration is longer, and fewer individuals survive through the stage.

To describe the time variation in through-stage survivorship, $S_{J}(t)$, in response to temperature variation, we again replace the integral $S_{J}(t)=e^{-\int_{t-\tau (t)}^{t}\;S[T(t)]dx}$ with its time derivative to obtain a DDE (Nisbet, 1997; Nisbet & Gurney, 1983):

(4) $$\frac{dS_{J}(t)}{dt}=S_{J}(t)\left[\frac{m_{J}[T(t)]d_{J}[T(t-\tau (t))]}{m_{J}[T(t-\tau (t))]}-d_{J}[T(t)]\right].$$

In the absence of density dependence, the long-term growth rate of a population is its intrinsic growth rate. For stage-structured populations, the intrinsic growth rate is given by the dominant eigenvalue of the Jacobian matrix for the system of equations depicting population dynamics. For the stage-structured model given by Equation (1), the temperature-dependent intrinsic growth rate in a constant thermal environment, that is, T(t)=T, is given by

(5) $$r(T)=-d_{A}(T)+\frac{1}{\tau (T)}W\left[b(T)\tau (T)e^{\tau (T)\left(d_{A}(T)-d_{J}(T)\right)}\right],$$

where W is the positive branch of the Lambert W function (Corless et al., 1996). The detailed derivation is given in Amarasekare and Coutinho (2013).

Importantly, Equation (5) allows us to derive the key properties of the fundamental thermal niche. The lower ($T_{min}$) and upper ($T_{max}$) temperature limits at which r(T)=0 constitute the lower and upper limits beyond which the population goes extinct. The temperature at which r(T) is maximized ($T_{{\mathrm{opt}}_{r}}$) is the temperature at which the species increases most quickly from low abundance.

### Sufficient condition for viability

The sufficient condition for viability is that the population be able to achieve a long-term (steady-state) abundance that is stable to perturbations. This requires population regulation via a DD feedback mechanism such as self-limitation (intraspecific competition).

#### Temperature dependence of self-limitation

There are two hypotheses regarding the temperature dependence of self-limitation. The first is based on temperature effects on activity levels. Because increasing activity levels increase the per individual demand for resources, and, assuming the resource supply remains approximately constant with temperature (Bernhardt et al., 2018; Savage et al., 2004), this hypothesis predicts that competition strength (quantified as per-capita competitive effect) should increase monotonically with increasing temperature according to the Boltzmann-Arrhenius relationship:

(6) $$q(T)=q_{TR}e^{A_{q}\left(\frac{1}{T_{R}}-\frac{1}{T}\right)},$$

where q(T) is the self-limitation strength at temperature $T,A_{q}$ is the Arrhenius constant, $T_{R}$ is the reference temperature as described earlier, and $q_{TR}$ is the self-limitation strength at the reference temperature (Appendix S1: Figure S1, red curve). Note that when T changes over time, as in Equations (1)–(4), the strength of self-limitation is also time dependent, given by q[T(t)].

The second hypothesis predicts that self-limitation should be strongest at temperatures optimal for reproduction because the demand for resources is likely to be most intense during periods of peak reproductive activity (Gao et al., 2013; Johnson et al., 2016). Now q(T) is unimodal and given by

(7) $$q(T)=q_{T_{\text{opt}}}e^{-\frac{{\left(T-T_{{\mathrm{opt}}_{\text{q}}}\right)}^{2}}{2{s_{q}}^{2}}},$$

where the strongest self-limitation $\left(q_{T_{\text{op}}}\right.$) occurs at temperature $T_{{\mathrm{opt}}_{\text{q}}}$, and $s_{q}$ depicts the temperature range over which self-limitation operates (Appendix S1: Figure S1, black curve).

#### Temperature-dependent population model with density dependence

Given these hypotheses, we can use a DD version of the DDE model given by Equations (1)–(4) to derive the sufficient condition for viability:

(8) $$\frac{dJ(t)}{dt}=B[T(t),A(t)]A(t)-M_{J}(t)-D_{J}[T(t),J(t)]J(t)\;\frac{dA(t)}{dt}=M_{J}(t)-D_{A}[T(t),A(t)]A(t)\;M_{J}(t)=B[T(t-\tau (t)),A(t-\tau (t))]\times A(t-\tau (t))\frac{m_{J}[T(t)]}{m_{J}[T(t-\tau (t))]}S_{J}(t)\;\frac{dS_{J}(t)}{dt}=S_{J}(t)\left[\frac{m_{J}[T(t)]D_{J}[T(t-\tau (t)),J(t-\tau (t))]}{m_{J}[T(t-\tau (t))]}-D_{J}[T(t),J(t)]\right]\;\frac{d\tau (t)}{dt}=1-\frac{m_{J}[T(t)]}{m_{J}[T(t-\tau (t))]},$$

where the terms and notation are the same as in the DI model, except that birth and mortality rates are now DD. The functions B[T(t),A(t)] and $D_{X}[T(t),X(t)]$, (X=J,A), describe the joint effects of temperature T(t) and density on per-capita birth and mortality rates. We depict DD birth and death rates using functions that empirical data show to be the most commonly observed density responses (Murdoch et al., 2003), that is, $B[T(t),A(t)]=b[T(t)]e^{-q_{b}[T(t)]A(t)}$ and $D_{X}[T(t),X(t)]=$ $d_{X}[T(t)]\left(1+q_{d_{X}}[T(t)]X(t)\right)$, where $q_{Y}[T(t)]$ is the temperature-dependent per-capita competition coefficient for trait Y(Y=b,d, corresponding to birth or death rate).

Because we are interested in applying the model to a Hemipteran insect system in which juvenile mortality is low and densities are highest at the adult stage, we focus our analyses on intraspecific density dependence operating on birth and adult mortality rates. However, the model is general and can be applied to any system in which density dependence occurs at either juvenile or adult stages.

#### Time to recover following a perturbation to steady-state abundance

We can solve the dynamical model (Equation 8) to obtain analytical expressions for steady-state abundance in a constant thermal environment (Appendix S1). In nature, populations are frequently perturbed from steady states by variation in the biotic and abiotic environment. It is therefore informative to also quantify how quickly a population might return to steady state following a perturbation. By computing the dominant eigenvalue of the Jacobian matrix of Equation (8), we can also obtain an analytical expression for the recovery time following a perturbation to the steady state:

(9) $$t_{\text{recovery}}(T)=\frac{-\lambda (T)}{\left|\lambda (T)\right|}\cdot \frac{1}{\left|\lambda (T)\right|},$$

where λ(T) is the dominant eigenvalue and |λ(T)| the absolute value of λ(T) (or its modulus when λ(T) is complex). Because a stable equilibrium occurs when the dominant eigenvalue has negative real parts, the negative sign in the numerator guarantees a positive recovery time when the equilibrium is stable. Analytical expressions for λ(T) under DD birth and mortality rates are given in Appendix S1 (Equations S3 and S5).

By incorporating mechanistic descriptions of life-history trait responses to temperature into the steady-state solutions, we can derive an analytical expression for the climate envelope, the range of temperatures over which an ectotherm species can maintain a viable long-term population under DD population regulation. By doing the same for the recovery time, we can get a trait-based prediction of how long it would take an ectotherm population to recover from a perturbation. The novelty of our approach is that it allows us to make predictions about temperature effects on long-term abundance and recovery from perturbations based solely on trait response data, completely independently of any population-level information.

We compare the climate envelope obtained using Equation (8) with the fundamental thermal niche derived using Equation (5) to determine whether the temperature at which long-term abundance is maximized under population regulation is higher or lower than the temperature at which the intrinsic growth rate is maximized (which occurs in the absence of population regulation; see Hypotheses and predictions below). Making this comparison requires that we first elucidate the temperature responses of the underlying life-history traits. We do this next.

## TEMPERATURE DEPENDENCE OF LIFE HISTORY

Phenotypic-level temperature responses of ectotherm life-history traits (per-capita birth, maturation, and mortality rates) are the result of temperature effects on the underlying biochemical processes (e.g., reaction kinetics and enzyme inactivation, hormonal regulation Johnson & Lewin, 1946; Kingsolver, 2009; Kingsolver et al., 2011; Nijhout, 1994; Ratkowsky et al., 2005; Schoolfield et al., 1981; Sharpe & DeMichele, 1977; van der Have, 2002; van der Have & de Jong, 1996). Temperature effects on rate-controlled processes such as reaction kinetics and enzyme inactivation yield phenotypic-level trait responses that are left-skewed or monotonically increasing/decreasing (Gillooly et al., 2001, 2002; Savage et al., 2004; van der Have, 2002; van der Have & de Jong, 1996). Mortality and maturation rates exhibit such responses (Amarasekare & Savage, 2012; Scranton & Amarasekare, 2017; Uszko et al., 2017). Temperature effects on regulatory processes such as neural and hormonal regulation (Hochachka & Somero, 2002; Long & Fee, 2008; Nijhout, 1994) yield symmetrically unimodal (e.g., Gaussian) trait responses at the phenotypic level. This is because regulatory processes are driven by negative feedbacks that push increasing and decreasing rate processes toward intermediate optima. Birth and attack rates exhibit such responses (Amarasekare & Savage, 2012; Englund et al., 2011; Scranton & Amarasekare, 2017; Uszko et al., 2017).

### Temperature response of mortality rate

The per-capita mortality rate of all ectotherms increases monotonically with temperature above the low temperature threshold for viability (see references in Gillooly et al., 2002; Savage et al., 2004). This response is well described by the Boltzmann-Arrhenius function for reaction kinetics (Gillooly et al., 2002; Savage et al., 2004; van der Have & de Jong, 1996; as in Figure 1b,d):

(10) $$d_{X}(T)=d_{X_{T_{R}}}e^{A_{d_{X}}\left(\frac{1}{T_{R}}-\frac{1}{T}\right)},$$

where $d_{X}(T)(X=J,A)$ is the mortality rate at temperature T (in degrees Kelvin); $A_{d_{X}}$ is the Arrhenius constant, which quantifies how fast the mortality rate increases with increasing temperature; and $T_{R}$ is a reference (baseline) temperature at which mortality is equal to $d_{X_{T_{R}}}$. The reference temperature occurs within the range where enzymes are 100% active. This is typically between 20 and $30^{{}^{\circ}}C$, with $24-25^{{}^{\circ}}C$ being the most common (Johnson & Lewin, 1946; Ratkowsky et al., 2005; Schoolfield et al., 1981; Sharpe & DeMichele, 1977). As with the temperature response of self-limitation (Equations 6 and 7), if temperature T changes in time, then so does the mortality rate: $d_{X}(T(t))$. This similarly holds for the birth and maturation rates explained below.

### Temperature response of birth rate

A large number of studies spanning a range of ectothermic taxa show that the per-capita birth rate exhibits a unimodal response to temperature (Amarasekare, 2015; Amarasekare & Savage, 2012; Carriere & Boivin, 1997; Dannon et al., 2010; Dell et al., 2011; Dreyer & Baumgärtner, 1996; Englund et al., 2011; Hou & Weng, 2010; Jandricic et al., 2010; Morgan et al., 2001), which is well-described by a Gaussian function (as in Figure 1a):

(11) $$b(T)=b_{T_{opt}}e^{-\frac{{\left(T-T_{{\mathrm{opt}}_{b}}\right)}^{2}}{2{s_{b}}^{2}}},$$

where $T_{{opt}_{b}}$ is the temperature at which the birth rate is maximal $\left(b_{T_{\text{opt}}}\right)$, and $s_{b}$ determines how quickly or slowly the response decays from the optimum. The latter provides a statistically quantifiable index of the response breadth (i.e., the temperature range over which the species can reproduce).

### Temperature response of maturation rate

The maturation rate of ectotherms exhibits a left-skewed temperature response (Kingsolver, 2009; Kingsolver et al., 2011; Schoolfield et al., 1981; Sharpe & DeMichele, 1977; van der Have, 2002; van der Have & de Jong, 1996) that results from the reduction in reaction rates at low and high temperature extremes due to enzyme inactivation. This response is well described by a thermodynamic rate process model (Ratkowsky et al., 2005; Schoolfield et al., 1981; Sharpe & DeMichele, 1977; as in Figure 1c):

(12) $$m_{J}(T)=\frac{\frac{T}{T_{R}}m_{TR}e^{A_{m}\left(\frac{1}{T_{R}}-\frac{1}{T}\right)}}{1+e^{A_{L}\left(\frac{1}{T_{L/2}}-\frac{1}{T}\right)}+e^{A_{H}\left(\frac{1}{T_{H/2}}-\frac{1}{T}\right)}},$$

where $m_{J}(T)$ is the maturation rate at temperature T (in degrees Kelvin); $m_{TR}$ is the maturation rate at the reference temperature $T_{R}$ at which the enzyme is 100% active; $A_{m}$, the enthalpy of activation divided by the universal gas constant R, quantifies temperature sensitivity; $T_{L/2}$ and $T_{H/2}$ are, respectively, the low and high temperatures at which the enzyme is 50% active; and $A_{L}$ and $A_{H}$ are the enthalpy changes associated with low- and high-temperature enzyme inactivation divided by R (Johnson & Lewin, 1946; Ratkowsky et al., 2005; Schoolfield et al., 1981; Sharpe & DeMichele, 1977; van der Have, 2002; van der Have & de Jong, 1996). When insufficient data exist to parameterize the full maturation function, alternative forms can be used. For example, when there are not enough data to fit the lower temperature limit of maturation, a form such as $m_{\text{alt}}(T)$, given in the note section of Table 1, can be used. If there is insufficient data at both the high and low ends of the maturation function, but maturation is expected to increase across the full range of temperatures for which reproduction occurs, an exponential function can be used such as

(13) $$m_{exp}(T)=m_{T_{R}}e^{A_{m}\left(\frac{1}{T_{R}}-\frac{1}{T}\right)}.$$

**FIGURE 2:**
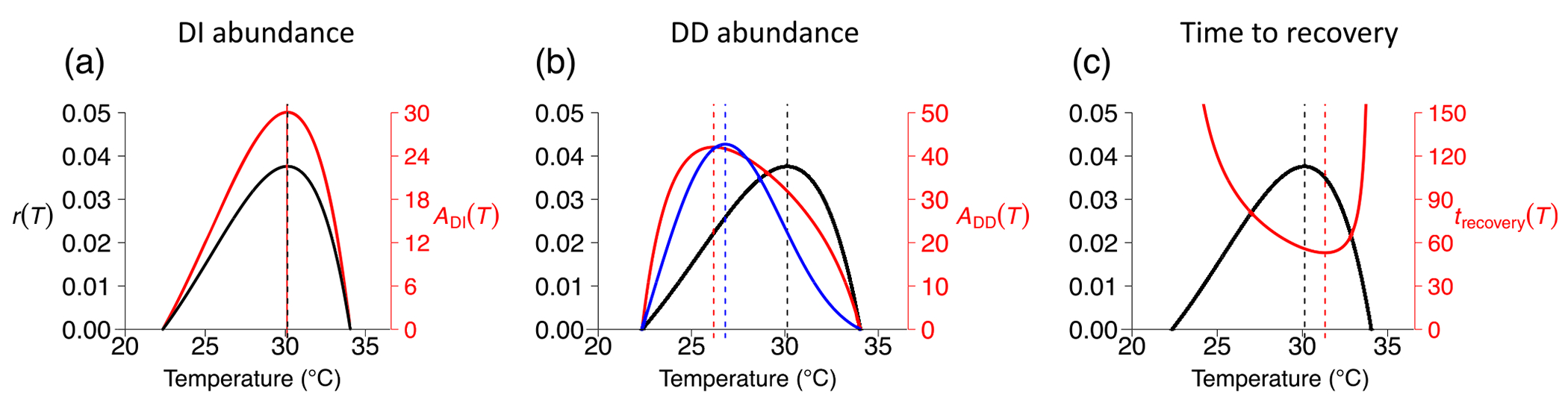
Model predictions for generic ectotherm species. (a) Temperature responses of intrinsic growth rate r(T) (black curve in all panels) and adult abundance $A_{DI}(T)$ (red curve) calculated from DI model (Equation 1). Note that the $A_{DI}(T)$ axis is in units of Log(adult individuals). (b) Comparison of temperature response of intrinsic growth rate (black curve) with those of steady-state adult abundance calculated from density-dependent (DD) model (Equation 8) for when competition affects adult mortality and temperature response of competition is monotonically increasing (blue) versus unimodal (red). The $A_{DD}(T)$ axis is in units of adult individuals. Dashed vertical lines indicate temperature at which abundance (blue, red) and r(T) (black) are maximized. (c) Comparison of intrinsic growth rate with recovery time to equilibrium calculated from DD model when density dependence operates on adult mortality. The strength of competition is monotonically increasing, but this does not affect stability (Appendix S1). Parameter values are realistic for warm-adapted species, such as those studied: $b_{T_{\text{opt}}}=2.2,T_{{\mathrm{opt}}_{b}}=302,s=3.4,T_{R}=297,d_{JT_{R}}=0.03,A_{d_{J}}=6260,d_{AT_{R}}=0.048,A_{d_{A}}=14,600,q_{TR}=q_{T_{\text{opt}}}=0.1,A_{q}=A_{d_{A}},T_{{opt}_{q}}=T_{{opt}_{b}},s_{q}=s,m_{T_{R}}=0.015$, and $A_{m}=11,500$. Maturation function Equation (13) was used.

We show an example using this form for a generic warm-adapted ectotherm species in Figure 2 (see Appendix S2 for details).

Several large-scale data analyses (Dell et al., 2011; Englund et al., 2011; Sunday et al., 2010) show that the qualitative nature of the trait responses described earlier (e.g., monotonic, left-skewed, Gaussian) is conserved across ectotherm taxa. This suggests that the trait-based models we develop here can be applied across a range of ectotherm species.

## METHODS

### Hypotheses and predictions

#### Temperature at which long-term abundance is maximized

We hypothesize that the temperature for which long-term abundance is maximized under DD population growth is lower than the temperature at which the intrinsic growth rate and abundance under DI growth are maximized. The rationale is as follows. Self-limitation should be strongest at temperatures for which abundance is maximized in the absence of competition ($T_{{\text{opt}}_{r}}$). When the strength of competition increases monotonically with increasing temperature, we expect strong competition at temperatures at and above $T_{{\text{opt}}_{r}}$. Therefore, a population under DD regulation should achieve maximal abundance at a temperature below $T_{{\text{opt}}_{r}}$. When competition strength is a unimodal function of temperature, maximum abundance cannot occur at a temperature above the optimal temperature for reproduction. This is because above the reproductive optimum, mortality rate increases and birth rate decreases with increasing temperature (Gillooly et al., 2002; Savage et al., 2004; van der Have & de Jong, 1996). Since r(T) is a composite of the temperature response of fecundity (which is symmetric unimodal) and the temperature responses of maturation and mortality (left-skewed and exponential, respectively) it is maximized at a temperature above the optimal temperature for reproduction (Amarasekare & Coutinho, 2013; Amarasekare & Savage, 2012). The key point is that regardless of whether the temperature response of competition is monotonic or unimodal, maximum abundance under DD regulation should occur at a temperature below $T_{\text{opt}t_{r}}$.

Testing this hypothesis requires comparing the temperature at which long-term abundance is maximized ($T_{\text{opt}}$) with the temperature at which the intrinsic growth rate is maximized ($\left.T_{{\text{opt}}_{r}}\right)$. When the thermal environment is constant (i.e., the species experiences the same temperature, on average, with few or no fluctuations around the mean), Equation (1) converges to a stable equilibrium. We solve for the steady-state solution when density dependence operates through birth or death rates and generate the climate envelope for when the temperature response of competition is monotonic or unimodal (Appendix S1). We make the connection between the intrinsic growth rate and maximum abundance when life-history traits are DI by simulating the DI model (Equation 2) long enough to produce a smooth function of abundances (expected for a dynamical model) over the full range of temperatures constituting the niche. This makes it possible to quantify an ectotherm species’ fundamental niche through measurements of its relative abundance.

#### Temperature effects on population recovery from perturbations

We expect the temperature at which the intrinsic growth rate is maximized to differ from the temperature at which the recovery time is minimized. We do so because the intrinsic growth rate (Equation 5) consists of the temperature responses of life-history traits only, while the dominant eigenvalue (Appendix S1: Equations S3 and S5) used to calculate the recovery time from a perturbation (Equation 7) includes temperature responses of life-history traits and competition.

### Testing theory with data

#### Natural history of insect community

We tested model predictions using trait response data from a naturalized-invasive insect community inhabiting the California Coastal Sage Scrub (CSS) community of Southern California. Both insects are Hemiptera (family: Pentatomidae), and both undergo five nymphal instar (nonreproductive) stages before reaching adulthood. The harlequin bug (*Murgantia histrionica*) is a naturalized herbivore that has inhabited the CSS in southern California for likely well over a century (Blatchley, 1934;Wallingford et al., 2011) and is adapted to the Mediterranean climate. The bagrada bug (*Bagrada hilaris*) is a recent introduction to North America and is native to subtropical and tropical regions of Southeastern Africa and South Asia (Hill, 2008). First recorded in Los Angeles County, California, in June 2008, the bagrada has since expanded across the southwestern United States and Mexico (Palumbo et al., 2016). Like the harlequin bug, bagrada is a generalist herbivore that feeds on a variety of crucifers (e.g., mustard, broccoli, cabbage). In the field, egg parasites are common for the harlequin, but neither bagrada or harlequin juveniles or adults incur mortality from natural enemies (Ludwig & Kok, 1998; Palumbo et al., 2016).

These naturalized and invasive insects represent an ideal system for testing predictions about the thermal niche and climate envelope because the two species are phylogenetically closely related (both are members of the family Pentatomidae) and have the same feeding niche (both are sap-sucking insects that predominantly feed on plants in the Brassicae family) but are adapted to different thermal regimes (subtropical vs. Mediterranean). We therefore expect them to differ in the characteristics of their fundamental thermal niche (e.g., $T_{min},T_{max}$, and $T_{\text{opt}}$).

#### Fitting mechanistic functions to trait response data: Experiments

We conducted laboratory experiments at different constant temperatures to quantify the two species’ response of life-history traits (birth, maturation, mortality) to temperature. We used bagrada individuals laid by first- and second-generation wild-caught individuals originating from collection sites in New Mexico (Tome and Las Cruces) and California (Leo Carrillo State Park and Lake Perris State Recreation Area) during summer 2013 (NM) and fall 2015 (CA) under California Department of Food and Agriculture State Plant Pest Movement Permit 2979 and California Department of Fish and Wildlife Scientific Collecting Permit 12788. We measured trait responses in temperature-controlled growth chambers (40±10% humidity; 12-h photoperiod) at the University of California, Los Angeles starting in fall 2013. Experiments were conducted at six temperatures (24, 27, 30, 33, 35, and $36^{{}^{\circ}}C$) and were started with newly laid eggs checked daily for first instar nymphal emergence. Emerged first instar nymphs were placed in a plastic cylinder vial (9.5cm in length by 2.8cm in diameter) containing a piece of cabbage approximately 2.5cm in diameter and sealed with a foam stopper. Individuals were checked daily for transition between each of the five juvenile life stages or death. Individuals who survived to the adult stage were put into mating pairs to measure lifetime fecundity. Cabbage was replaced in both nymphal and adult containers every 48–72 h. Data for the harlequin bugs, collected at eight temperatures (15, 18, 21, 24, 27, 29, 33, and $35^{{}^{\circ}}C$) using the same resource and similar protocols, are reported in Amarasekare and Savage (2012) and Amarasekare and Sifuentes (2012). Both bagrada and harlequin data are available in Simon and Amarasekare (2024a).

For both species, per-capita birth rate was measured as the number of eggs laid per adult life span of a given female and per-capita mortality rate, the inverse of time until death, where time until death is number of days from first instar nymph emergence until juvenile death (juvenile mortality) or from the date of adult emergence until death (adult mortality). Per-capita maturation rate was measured as the inverse of juvenile development duration (τ), which is the number of days from first instar emergence to fifth molt (the molt from which the adult emerges).

#### Fitting mechanistic functions to trait response data: Parameter estimation

We followed previous studies (e.g., Amarasekare & Savage, 2012; Lin et al., 2023; Scranton & Amarasekare, 2017) in quantifying the mean value of each trait by averaging over the number of replicates (individuals) at each experimental temperature. This approach allows one to estimate the standard error and probability associated with each parameter estimate and, hence, the reliability of the estimates. We fitted mechanistic temperature response functions (Equations 10–12) to these mean trait values using least-squares nonlinear regression. Fits were conducted with the “nls” function of the base stats package in R version 4.2.2 (R Core Team, 2017). This analysis assumes Gaussian error around predictions of population means, a reference trait value measured at a reference temperature typically determined by the investigator (given in Table 1 as “$T_{R}=$…” for applicable parameters), and that parameter ranges are dictated by biological realism (i.e., the low temperature at which an enzyme is 50% active must be greater than the freezing temperature 273K). Table 1 gives the resulting parameter estimates and Figure 1a–d depict the observed and fitted trait response functions for the two species. When the nls algorithm did not converge due to a lack of data at the extremes, as was the case with maturation data for the bagrada at high temperatures, biologically realistic values were assigned. However, comparison with an exponential function (Equation 13) that does not incorporate the high temperature decline shows a negligible difference in model predictions (Appendix S2). The code used for parameter estimation is available in Simon and Amarasekare (2024b).

#### Analysis of dynamical models

We conducted two analyses. First, we used DI (Equations 1–4) and DD (Equation 8) models with realistic parameter values for ectotherm species to test the general predictions made in *Hypotheses and predictions* above. We then parameterized the models with trait response data for the two insect species to test the validity of model predictions when applied to real species. In both cases, we used the DI model to quantify the temperature response of the intrinsic growth rate and climate envelope in a constant thermal environment. Since the DI model has no long-term equilibrium, we simulated exponential growth using the dde command of the PBSddesolve package (version 1.13.3; Couture-Beil et al., 2023) in R (R Core Team, 2017) and long-term abundance at the end of 5 years (long enough to achieve a stable-stage distribution across the thermal niche) for a range of mean habitat temperatures (see Simulation_FigureGeneration_Rcode.R in Simon & Amarasekare, 2024b). This gives the species’ climate envelope in the absence of population regulation. We compared the temperature responses of intrinsic growth rate and DI climate envelope to test whether the prediction that when population growth is DI, the temperature at which abundance is maximized coincides with the temperature at which r(T) is maximized.

We used the DD model to generate the climate envelope under population regulation for the same range of mean temperatures as for the DI model. We compared the temperature responses of intrinsic growth rate and DD climate envelope to test the prediction that the temperature at which abundance is maximized under DD growth is lower than that at which r(T) is maximized. We calculated the recovery time following a perturbation under DD growth using Equation (7) to test the expectation that the temperature at which the intrinsic growth rate is maximized should be different from the temperature at which the recovery time is minimized.

We calculated the degree of thermal niche overlap between the two insect species by integrating r(T) for each species at their respective thermal limits for viability:

(14) $$\int_{T_{r_{{\text{bagrada}}_{\text{min}}}}}^{T_{\mathrm{interest}}}r_{\text{bagrada}}(T)dT+\int_{T_{\mathrm{interest}}}^{T_{r_{{\text{harlequin}}_{\text{max}}}}}r_{\text{harlequin}}(T)dT,$$

where $T_{r_{{\text{bagrada}}_{\min}}}$ and $T_{r_{{\mathrm{harlequin}}_{\max}}}$ are, respectively, the lower and upper temperature limits for the viability of the bagrada and harlequin bugs ($T_{min}$ and $T_{max}$ in Table 2). The intersection of the thermal niches of the two species, $T_{\text{intersect}}$, occurs when $r_{\text{bagrada}}(T)-$ $r_{\text{harlequin}}(T)\approx 0$ (equivalently, where $r_{\text{bagrada}}(T)\approx$ $\left.r_{\text{harlequin}}(T)\right)$. We solved for the intersection with accuracy $r_{\text{bagrada}}(T)-r_{\text{harlequin}}(T)\leq 10^{-6}$ by calculating the difference at successively smaller T intervals. Equation (14) was calculated using the “Nintegrate” function in Mathematica (Wolfram Research, Inc, 2019). The code is available in Simon and Amarasekare (2024b).

## RESULTS

### General predictions from dynamical models

As expected, the temperature at which the intrinsic growth rate, r(T), was maximized was indeed the same temperature at which abundance was maximized (Figure 2a). This suggests that, in the absence of intraspecific competition, the temperature at which an ectotherm species’ ability to increase when rare is the one at which it also reaches the highest abundance. As also expected, the temperature at which equilibrium abundance was maximized was lower than the temperature at which the intrinsic growth rate was maximized (Figure 2b). We found that the temperature at which recovery time was minimized (the temperature at which recovery from a perturbation was the fastest) was higher than that at which the intrinsic growth rate was maximized (Figure 2c). The key point is that steady-state abundance is maximal at the cooler end of the thermal niche, while the fastest response to perturbation occurs at the warmer end of the niche. These outcomes ensue regardless of the life-history trait (fecundity, mortality) on which density dependence operates or the qualitative form of the temperature response of competition (monotonic vs. unimodal; Appendix S1: Figure S2).

### Testing theory with data from a naturalized–invasive insect community

**FIGURE 3:**
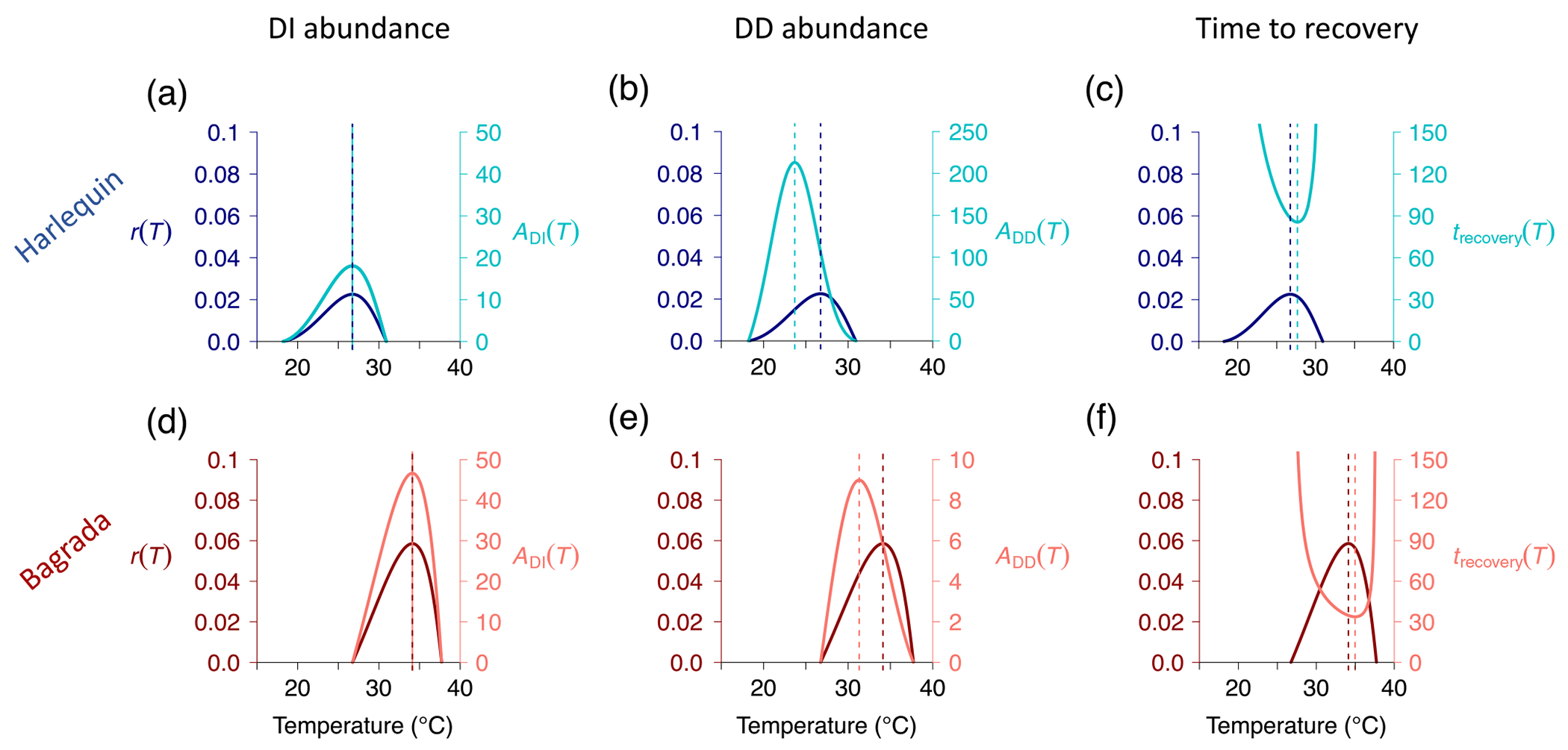
Temperature-dependent intrinsic growth rate, r(T), and climate envelope under density-independent (DI), $A_{DI}(T)$, and density-dependent (DD) population growth, $A_{DD}(T)$, for the naturalized harlequin bug (top; blue) and invasive bagrada (bottom; red). Panels (a) and (d) depict the intrinsic growth rate (dark blue curve in [a], dark red curve in [d]) and adult abundance (in units Log[adult individuals]) under DI growth (light blue curve in [a], light red curve in [d]). Panels (b) and (e) compare r(T) with $A_{DD}(T)$ when competition affects adult mortality via a monotonic temperature response of competition. Units of $A_{DD}(T)$ are adult individuals. Panels (c) and (f) depict r(T) and the recovery time to equilibrium $t_{\text{recovery}}(T)$ (in units of time). Parameters: $q_{TR}=0.1$ for both bugs; $A_{q}=A_{d_{A}}$ for each respective bug; all other parameters are given in Table 1.

Analyses of the parameterized models confirm that our predictions hold when applied to insect species in the wild. When population growth is DI, the temperature of maximum abundance coincides with the temperature at which r(T), and hence the ability to increase when rare, is maximized (Figure 3a,d). This occurs at $34.1^{{}^{\circ}}C$ in the warm-adapted bagrada and, almost $7^{{}^{\circ}}C$ degrees cooler, at $26.7^{{}^{\circ}}C$ in the cooler-adapted harlequin (Table 2).

As predicted, when population growth was DD, the temperature at which abundance was maximized was lower than that at which the intrinsic growth rate r(T) was maximized (Figure 3b,e). Interestingly, we found that the temperature at which the bagrada achieved its maximum abundance (28.9 and $31.3^{{}^{\circ}}C$, DD fecundity, mortality, respectively; Figure 3e) was very close to the temperature at which the harlequin’s intrinsic growth rate was maximized $\left(26.7^{{}^{\circ}}C\right.$ Figure 3a). Additionally, bagrada’s maximum abundance also occurred very close to the temperature for which harlequin’s time to recovery was shortest (29.2 and $27.7^{{}^{\circ}}C$, DD fecundity, mortality, respectively; Figure 3c).

**FIGURE 4:**
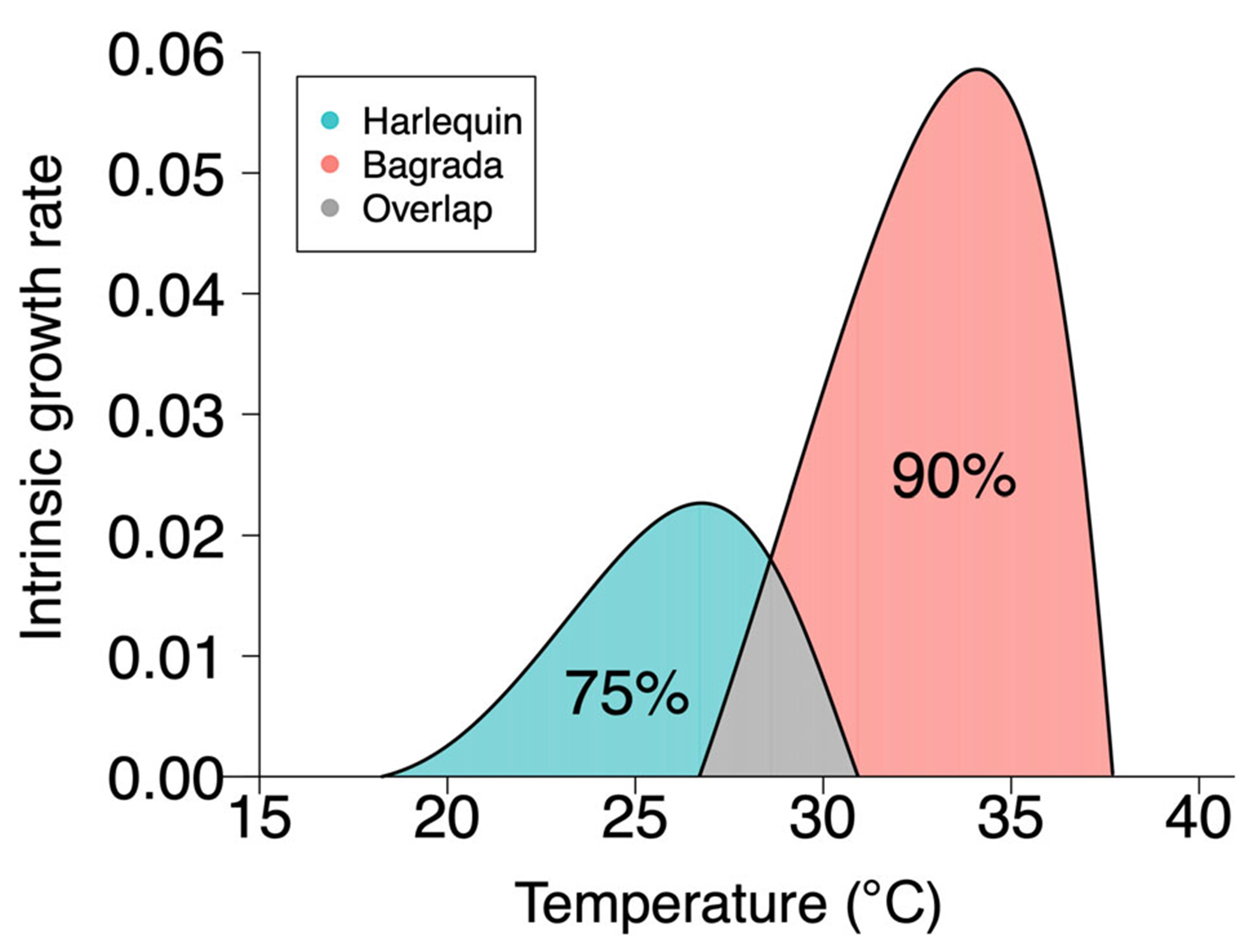
Fundamental thermal niches of naturalized harlequin (blue and gray regions) and invasive bagrada bugs (red and gray regions) as predicted from trait response data (Figure 1, Table 1). Niche overlap is shown in gray. Niche metrics are given in Table 2.

Lower and upper thermal limits of the fundamental niche are greater for the bagrada, which is of subtropical origin $\left(T_{min}=26.8^{{}^{\circ}}C,T_{max}=37.7^{{}^{\circ}}C\right.$), than for the Mediterranean-adapted harlequin bug ($T_{min}=18.2^{{}^{\circ}}C$, $T_{max}=30.9^{{}^{\circ}}C$). The two species’ niches overlap within the temperature range $26.8–30.9^{{}^{\circ}}C$ (Figure 4, Table 2). This overlap constitutes ~25% of the harlequin’s thermal niche but only ~10% of the bagrada bug’s niche (see NicheOverlap_MathematicaCode_OUTPUT.pdf in Simon & Amarasekare, 2024b). Importantly, this temperature range of niche overlap includes the temperatures at which the harlequin bug's intrinsic growth rate $\left(26.7^{{}^{\circ}}C\right)$ and recovery time are maximized (29.2 and $27.7^{{}^{\circ}}C$ for DD fecundity, mortality, respectively) and the temperature at which the bagrada’s steady-state abundance is maximized when density dependence operates on fecundity $\left(28.9^{{}^{\circ}}C\right.$; the maximum under DD mortality slightly exceeds niche overlap at $31.3^{{}^{\circ}}C$).

## DISCUSSION

Climate warming is widely expected to shift species’ distributions, enhance extinction risk, and increase the invasion of exotic pests and pathogens. Predicting the ecological impacts of climate warming requires that we understand how temperature effects on ectotherm species’ life-history traits translate into population-level patterns of abundance and distributional changes through the shaping of species’ thermal niches. Here we developed a mechanistic, trait-based framework for characterizing the fundamental thermal niche and, hence, a species’ climate envelope (the range of temperatures over which a species maintains a viable population), based on how species’ life-history traits and population regulatory mechanisms respond to temperature (Figure 1).

The particular strength of our framework is that it is sufficiently biologically realistic to apply to specific species but is, at the same time, sufficiently general to apply broadly across ectotherm taxa. Its novelty is that it generates testable predictions about species’ abundances and distributions based solely on information on the temperature responses of the underlying life-history traits. The predictions are therefore completely independent of observed distribution and abundance patterns. This is important because, when a framework is used based on species’ traits for which the temperature responses can be carefully measured in the laboratory, the thermal niche can be quantified in the absence of biotic filters that generally plague measurements obtained from the field. This allows for more accurate predictions of climate envelopes of ectotherm species of interest. Further, several large-scale data analyses show that the qualitative nature of the temperature responses of life-history traits (e.g., monotonic, left-skewed, Gaussian) is conserved across ectotherm taxa (Kingsolver, 2009; Kingsolver et al., 2011; van der Have, 2002; van der Have & de Jong, 1996) and their parameters are thermodynamically constrained to lie within narrow limits (Brown et al., 2004; Dell et al., 2011; Gillooly et al., 2001, 2002; Savage et al., 2004). This affords the advantage of being able to use parameter values of related species from similar thermal environments when data are unavailable for a species for whom a climate envelope is required.

Data limitation is most likely to occur when quantifying the temperature response of the maturation rate. Most previous studies quantified only the rising portion of the maturation curve, that is, the temperature range within which the maturation rate increases exponentially with temperature (Equation 13; Appendix S2: Figure S1, solid curve; see references in Gillooly, 2002). This is largely because the metabolic theory of ecology (Brown, 2004; Savage et al., 2004), the prevailing framework for temperature dependence at the time, did not consider the high temperature decline in their formulations. The advent of climate warming has necessitated a broadening of the framework for characterizing trait response functions. Given the importance of climate warming in driving species distributions, future empirical studies should concentrate on quantifying the maturation rate at high temperature extremes so that the decline at these extremes can be quantified. Based on our experience, extending the temperature measurements to four or five temperature treatments above the optimal temperature for reproduction ($T_{{\text{opt}}_{b}}$ in Equation 11) should suffice to obtain statistically significant fits to the two parameters that characterize the high temperature decline (Equation 12; Ardelan et al., 2023; Scranton & Amarasekare, 2017).

The framework we present applies broadly across multicellular ectotherm species despite life-history differences in the number of pre-reproductive stages. Such differences do not require modification to the model when density dependence acts only on adults or on all juvenile stages equally because pre-reproductive (juvenile) stages then simply act as a time lag (Murdoch et al., 2003). In systems for which density dependence acts at some but not all juvenile stages, the juvenile class in Equations (1) and (8) can be split into multiple juvenile classes, each with its own corresponding equation (e.g., Johnson et al., 2016).

Our framework is sufficiently general to apply to abiotic factors other than temperature (e.g., humidity, salinity) so long as the response of life-history traits and self-limitation can be measured at different magnitudes of the abiotic condition of interest. It is also feasible to make predictions based on the joint effects of multiple abiotic factors by modifying the existing framework, which is an important area for future research. Quantifying the temperature response (or other abiotic response) of life-history traits of long-lived or very large ectotherms may not be possible in lab-controlled settings. For these cases, the use of well-controlled field settings using large numbers per replicate could be a viable alternative. Research into this possibility is needed.

The key innovation of our framework is the development of metrics (intrinsic growth rate, abundance, and recovery time as a function of temperature) that can be used to generate thermal range plots for native and invasive species. These allow us to predict, a priori, the likelihood of competitive interactions and the proportion of each species’ range within which such interactions are likely to occur. These thermal plots can be compared with temperature–abundance data from the field to determine where a species could be but is absent due to interactions with competitors and/or natural enemies or due to dispersal limitation and to where it could potentially expand under various scenarios of climate warming. By characterizing both necessary and sufficient conditions for population viability in terms of species abundances, we are able to obtain a complete description of the fundamental niche under both DI (e.g., invasive species in their initial establishment phase) and DD (e.g., native species’ typical dynamics) population growth.

Our trait-based framework yields two important insights into the fundamental thermal niche of ectotherms. First, maximum abundance occurs at warmer temperatures in populations experiencing DI growth compared to populations experiencing DD growth. Second, the type of population growth determines the degree of congruence between the temperature at which abundance is maximized and the temperature at which the species can increase most quickly from low abundance. In populations exhibiting unbounded growth, the temperature at which abundance is maximized is the same as the temperature at which the species can increase most quickly from low abundances; in populations exhibiting bounded growth, the temperature at which abundance is maximized is lower than the temperature at which the species can increase most quickly from low abundances. Importantly, these are general outcomes that prevail regardless of which life-history trait density dependence operates on or the qualitative nature (monotonic vs. unimodal) of the temperature response of intraspecific competition. They suggest that climate warming will have differential effects on native or naturalized versus invasive species. For instance, the fact that the temperature of maximum abundance and temperature of fastest recovery from low abundances coincide suggests an advantage of climate warming for species exhibiting unbounded growth (e.g., crop pests, newly introduced species). Similarly, the fact that the temperature of maximum abundance is lower than the temperature of fastest recovery suggests a disadvantage for species exhibiting bounded growth (e.g., native species well-established in their habitats). Putting this in the context of interactions between native and invasive species, their ability to increase when rare at warmer temperatures at which native species exhibit lower abundances and, hence, weaker competitive pressure give invasive species a greater advantage in establishing in newly colonized habitats. The important implication for native species, which is particularly relevant when generating climate envelopes for such species, is that the temperature at which one is likely to observe highest abundance in the field is not the temperature that is optimal for reproduction and population growth, but the one at which effects of self-limitation are minimal.

Our findings support the widespread expectation that climate warming will increase the spread of invasive pests and pathogens. An increase in the environmental temperature at a given location will draw native species away from the cooler temperatures at which their abundance and competitive pressure on invasive species are greatest. At the same time, it will subject the invasive species to the warmer temperatures at which their ability to increase when rare is greater. The faster an exotic species can increase when thermal conditions are favorable, the greater the likelihood of its successful establishment. The advantage of our framework is that we can predict, a priori, which native species will be at greater risk and which invasive species have the greater advantage, based on how their life-history traits respond to temperature. It is customary for entomologists and pest management specialists to quantify temperature effects on the life-history traits of pests and newly invaded species through laboratory experiments. In fact, the pest management and entomological literature is replete with such studies (e.g., Correa et al., 2021; Karpicka-Ignatowska et al., 2021; Sun et al., 2022), meaning that the information required for quantifying the thermal niche of invasive species is likely to be widely available.

Tests of our model predictions with data for the naturalized (harlequin bug) and invasive species (bagrada) confirm the key conceptual insights of our trait-based framework. First, when population growth is DI, the temperature at which abundance is maximized coincides with the temperature at which r(T), and hence the ability to increase when rare, is maximized. Second, when population growth is DD, the temperature at which abundance is maximized is lower than that at which the intrinsic growth rate, r(T), is maximized. Third, the range of thermal niche overlap between the two species includes the temperatures for which the harlequin bug’s intrinsic growth rate and recovery time are maximized and the temperature at which the bagrada’s abundance (under DD population growth) is maximized. Interestingly, we found that the bagrada achieved its maximum abundance at a temperature very close to that for which the harlequin’s recovery time was the shortest, suggesting that competitive interactions with the invasive bagrada could impact the naturalized harlequin’s ability to respond to external perturbation. We additionally found that the bagrada achieved its maximum abundance at a temperature close to that at which the harlequin bug’s intrinsic growth rate was maximized. In contrast, bagrada's intrinsic growth rate was maximized at a temperature that was above the upper temperature limit for the harlequin bug’s viability and was very close to the maximum temperature observed in the CSS habitat $\left(\sim 36^{{}^{\circ}}C\right.$ in the University of California San Joaquin Marsh Reserve, Irvine, CA, USA; Simon, 2017). The upper temperature limit for bagrada’s viability is slightly above the maximum observed CSS temperature.

The ability to increase when rare is a critical component of invasion success. However, if the temperature at which a species’ ability to increase when rare is the greatest coincides with the temperature at which its competitor’s abundance is maximized, invasibility will be hindered by strong competition from the resident species. Since the harlequin bug’s (the resident species) abundance is maximized at a temperature much lower than that at which the bagrada’s (the invasive species) ability to increase when rare is the greatest, one would expect the bagrada’s invasion of the CSS community to be relatively unhindered by competitive pressure from the harlequin bug. Had their roles been reversed (i.e., bagrada had been the naturalized species), the harlequin bug would have had difficulty increasing from initially small numbers because the temperature at which it has the greatest ability to increase when rare is also the temperature at which competitive pressure from bagrada is the greatest (at least when density dependence acts on fecundity).

This finding illustrates an interesting asymmetry between species in their invasion success based on their latitudinal origin. Invasion by an exotic species is most likely to succeed when the temperature at which the exotic species’ intrinsic growth is maximized (i.e., the temperature at which its ability to increase when rare is the greatest) is greater than the temperature at which the native (or naturalized) species’ abundance is maximized. An ectotherm species of Mediterranean or temperate origin introduced to a tropical habitat may be at a disadvantage because the temperature at which its ability to increase when rare is likely to coincide with the temperature at which the native species is the most abundant (and, hence, exerts the strongest competitive pressure on an incoming species). In contrast, an ectotherm species of tropical origin introduced to a Mediterranean or temperate habitat is likely to have greater invasion success because the temperature at which its invasibility is the greatest is likely to be higher than the temperature at which competitive pressure from the native species is the strongest. Such a directionality in invasion success, with tropical species having greater success in invading temperate habitats, has been reported based on data of both extant and extinct species (Jablonski et al., 2006, 2013), which recent theory (Amarasekare & Johnson, 2017; Amarasekare & Simon, 2020) attributes to tropical species having higher optimal temperatures for reproduction and lower mortality during temperate summers compared to temperate species. An interesting future direction would be to conduct a broader analysis, based on available data from the literature, to determine whether warm-adapted invasive species from lower latitudes have an intrinsic advantage when interacting with cold-adapted native species from higher latitudes.

## Supplementary Material

Appendix S1

Appendix S2

## Figures and Tables

**TABLE 1:** Parameter estimates (±SE) for temperature responses of life-history traits of bagrada and harlequin bugs fitted from data.

Life-history trait	Species							
	Bagrada bug				Harlequin bug			
	Estimate ± SE	*t* value	*p*-value	df	Estimate ± SE	*t* value	*p*-value	df
Birth rate b(T)								
$b_{T_{opt}}$	3.586±1.033	3.471	$7.39\times 10^{-2}$	2	0.8921±0.1119	7.973	p<0.001	5
*T*_opt_*b*__	306.6±1.29	238.24	*p*<0.001	2	298.3±0.41	722.292	*p*<0.001	5
s	3.659±1.617	2.264	0.152	2	3.085±0.468	6.598	$1.2\times 10^{-3}$	5

Maturation rate m(T)								
$m_{T_{R}}$	$0.0168^{a}\left(T_{R}=297\right)$	NA	NA	NA	$0.0138\left(T_{R}=297\right)$	NA	NA	NA
$A_{m}$	$10,671.0\pm 728.2^{a}$	14.65	p<0.001	3	13,480±592.6	22.75	$1.93\times 10^{-3}$	2
*A_L_*	NA^a^	NA	NA	NA	−100,000^b^	NA	NA	NA
*A_H_*	90,000^b^	NA	NA	NA	48,150±4223	11.40	7.6×10^−3^	2
*T_L/2_*	NA^a^	NA	NA	NA	273^b^	NA	NA	NA
$T_{H/2}$	$312^{b}$	NA	NA	NA	303.8±0.197	1544.93	p<0.001	2

Juvenile mortality rate $d_{J}(T)$								
$d_{JT_{R}}$	$0.0287\left(T_{R}=297\right)$	NA	NA	NA	$0.0343\left(T_{R}=297\right)$	NA	NA	NA
$A_{d_{J}}$	6779.1±720.5	9.409	$2.54\times 10^{-3}$	3	5743±1516	3.787	0.0193	4

Adult mortality rate $d_{A}(T)$								
$d_{A_{T_{R}}}$	$0.0926\left(T_{R}=297\right)$	NA	NA	NA	$0.0029\left(T_{R}=297\right)$	NA	NA	NA
$A_{d_{A}}$	12,355.6±552.6	22.36	p<0.001	3	16,824±705	23.86	p<0.001	6

*Note*: Estimates for life-history trait parameters were conducted using nonlinear least squares. Temperatures are in degrees Kelvin.

a Given insufficient data to quantify the low temperature decline, a simpler model, $m_{\mathrm{alt}}(T)$, was used to fit the bagrada maturation rate data: $m_{\mathrm{alt}}(T)=\frac{\frac{T}{T_{R}}m_{T_{R}}e^{A_{m}(\frac{1}{T_{R}}-\frac{1}{T})}}{1+e^{A_{H}(\frac{1}{T_{H/2}}-\frac{1}{T})}}$.

b This was a biologically reasonable choice.

**TABLE 2:** Parameters characterizing temperature dependence of intrinsic growth rate, long-term abundance, and recovery time for invasive bagrada bug and naturalized harlequin bug.

Metric	Species	
	Bagrada	Harlequin
Intrinsic growth rate r(T)		
$T_{{opt}_{r}}\left({}^{\circ}C\right)$	34.1	26.7
$r_{T_{\text{opt}}}$ (per day)	0.058	0.023
$T_{min}\left({}^{\circ}C\right)$	26.8	18.2
$T_{max}\left({}^{\circ}C\right)$	37.7	30.9

Density-dependent fecundity		
$T_{{\text{opt}}_{A_{\text{DD}}}}\left({}^{\circ}\text{C}\right)$	28.9	19.7
$A_{DD}\left(T_{opt}\right)$ (adult individuals)	18.1	62.6
$T_{{\mathrm{opt}}_{t_{\mathrm{recovery}}}}({}^{\circ}C)$	35.2	29.2
$t_{{\mathrm{recovery}}_{T_{\mathrm{opt}}}}(\mathrm{days})$	0.451	0.029

Density-dependent mortality		
$T_{{\text{opt}}_{A_{\text{DD}}}}\left({}^{\circ}\text{C}\right)$	31.3	23.7
$A_{DD}\left(T_{\mathrm{opt}}\right)$ (adult individuals)	8.98	213.2
$T_{{\text{opt}}_{t_{\text{recovery}}}}\left({}^{\circ}\text{C}\right)$	35.0	27.7
$t_{{\text{recovery}}_{T_{\text{opt}}}}$ (days)	0.029	0.012

## Data Availability

Data (Simon & Amarasekare, 2024a) are available in Dryad at https://doi.org/10.5061/dryad.rxwdbrvfp. Code (Simon & Amarasekare, 2024b) is available in Zenodo at https://doi.org/10.5281/zenodo.8245092.
