# Supplementary material for: Predicting the fundamental thermal niche of ectotherms
Source: Ecology. Author manuscript; Available in PMC 2025 May 1. (PMC11374413; doi:10.1002/ecy.4289)
Supplement: Appendix S1 [file NIHMS1976892-supplement-Appendix_S1.pdf]

Margaret W. Simon, Priyanga Amarasekare
Predicting the fundamental thermal niche of ectotherms
Supplementary Materials for Article in *Ecology*

This file includes:

Appendix S1: Main text including Eqs. (S1)-(S5)
Appendix S1: References
Appendix S1: Figures S1 and S2

Appendix S1. Long-term outcomes of the stage-structured delay model in a constant thermal environment

DENSITY-INDEPENDENT POPULATION GROWTH

In a constant thermal environment (i.e., the species experiences the same temperature, on average, over the year: $T(t) \approx T$), Eqs. (1) – (4) in the main text simplify to:

$$\frac{dJ(t)}{dt} = b(T)\,A(t) - b(T)\,A\big(t-\tau(T)\big)\,e^{-d_J(T)\,\tau(T)} - d_J(T)\,J(t)$$
$$\frac{dA(t)}{dt} = b(T)\,A\big(t-\tau(T)\big)\,e^{-d_J(T)\,\tau(T)} - d_A(T)\,A(t) \qquad (S1)$$

where the functions $b(T)$, $d_X(T)$ ($X = J, A$) depict the density-independent per capita birth and mortality rates at temperature T, Eqs. (11) and (10) in the main text, respectively. Since maturation and mortality rates are no longer time-dependent, development delay (τ) and juvenile survivorship (S) do not vary over time and are equal to, respectively, $\frac{1}{m_J(T)}$ and $e^{-d_J(T)\,\tau(T)}$. The function $m_J(T)$ gives the density-independent per capita maturation rate at temperature T.

We can compute the long-run growth rate as the dominant eigenvalue of Eq. (S1), which gives Eq. (5) in the main text.

DENSITY-DEPENDENT POPULATION GROWTH

When population growth is density-dependent, we can solve the dynamical model (Eq. (8) in the main text) for analytical expressions of steady-state abundance when the thermal environment is constant. We focus on the empirically common cases for which intraspecific competition affects either fecundity or adult mortality.

Intraspecific competition affects fecundity

When density-dependence operates on fecundity, the per capita birth rate is a decreasing function of density. We assume the form $B[T(t), A(t)] = b\big(T(t)\big)\,e^{-q(T(t))\,A(t)}$. In a constant thermal environment, this gives us the following version of Eq. (8):

$$\frac{dJ(t)}{dt} = b(T)\,A(t)\,e^{-q(T)\,A(t)} - M_J(t) - d_J(T)\,J(t)$$
$$\frac{dA(t)}{dt} = M_J(t) - d_A(T)\,A(t) \qquad (S2)$$

$$M_J(t) = b(T)\, A\big(t - \tau(T)\big)\, e^{-q(T)\, A(t-\tau(T))}\, e^{-d_J(T)\, \tau(T)}$$

where the functions $b(T)$, $d_X(T)$ ($X = J, A$) and $M_J(T)$ again depict the density-independent per capita birth, mortality and maturation rates at temperature T (Eqs. (11), (10), and (12), respectively). As in the density-independent model, development delay (τ) and juvenile survivorship (S) do not vary over time and are equal to $\frac{1}{m_J(T)}$ and $e^{-d_J(T)\, \tau(T)}$.

At equilibrium $\frac{dJ(t)}{dt} = 0$, $\frac{dA(t)}{dt} = 0$, $J(t) = J\big(t - \tau(T)\big) = J^*$ and $A(t) = A\big(t - \tau(T)\big) = A^*$. The non-trivial equilibrium is given by $J^* = \frac{dA(T)}{d_J(T)} \left[\exp\left(\frac{d_J(T)}{m_J(T)}\right) - 1\right] A^*$ and $A^* = \frac{\ln\left(\frac{b(T)}{d_A(T)}\right) - \frac{d_J(T)}{m_J(T)}}{q(T)}$. Note that the developmental delay is expressed in terms of the maturation rate, i.e., $\tau(T) = \frac{1}{m_J(T)}$.

In a constant thermal environment, the equilibrium is a function of the strength of competition ($q(T)$) but the stability of the equilibrium is not (Nisbet and Gurney, 1983; Nisbet, 1997; Murdoch et al., 2003). The dominant eigenvalue ($\lambda(T)$) of the Jacobian of Eq. (S2) evaluated at the non-trivial equilibrium is given by:

$$\lambda(T) = -d_A(T) + \frac{1}{\tau(T)} W\left[b(T)\, \tau(T)\, e^{\boldsymbol{A(T)}}\, \big(1 + \boldsymbol{F(T)}\big)\, e^{\tau(T)\left(d_A(T) - d_J(T)\right)}\right] \qquad (S3)$$

where $\boldsymbol{F(T)} = -\ln\left(\frac{b(T)}{d_A(T)}\right) + d_J(T)\, \tau(T)$.

Intraspecific competition affects adult mortality

When intraspecific competition affects adult mortality in a constant thermal environment we have the following version of Eq. (8) in the main text:

$$\frac{dJ(t)}{dt} = b(T)\, A(t) - M_J(t) - d_J(T)\, J(t)$$
$$\frac{dA(t)}{dt} = M_J(t) - d_A(T)\, \big(1 + q(T)\, A(t)\big)\, A(t) \qquad (S4)$$
$$M_J(t) = b(T)\, A\big(t - \tau(T)\big)\, e^{-d_J(T)\, \tau(T)}$$

where we assume the form $D[T(t), A(t)] = d_A\big(T(t)\big)\big(1 + q(T)\, A(t)\big)$. The non-trivial equilibrium is $J^* = \frac{b(T)\, A^*\left(1 - e^{-d_J(T)\, \tau(T)}\right)}{d_J(T)}$ and $A^* = \frac{b(T) e^{-d_J(T)\, \tau(T)} - d_A(T)}{d_A(T)\, q(T)}$. The dominant eigenvalue is:

$$\lambda(T) = -d_A(T) + \boldsymbol{C(T)} + \frac{1}{\tau(T)} W\left[b(T)\, \tau(T) e^{\tau(T)\left(d_A(T) - d_J(T) - \boldsymbol{C(T)}\right)}\right] \qquad (S5)$$

where $\boldsymbol{C(T)} = 2\big(d_A(T) - b(T)\, e^{-d_J(T)\, \tau(T)}\big)$.

References

Nisbet RM and Gurney W. 1983. The systematic formulation of population-models for insects with dynamically varying instar duration. Theoretical Population Biology 23: 114–135.

Nisbet R. 1997. Delay-differential equations for structured populations. In: Tuljapurkar S and Caswell H (Eds.) Structured-population models in marine, terrestrial, and freshwater systems, 89–116. Chapman and Hall, New York.

Murdoch W, Briggs CJ, and M NR. 2003. Consumer resource dynamics. Princeton University Press, Princeton New Jersey.

Figures

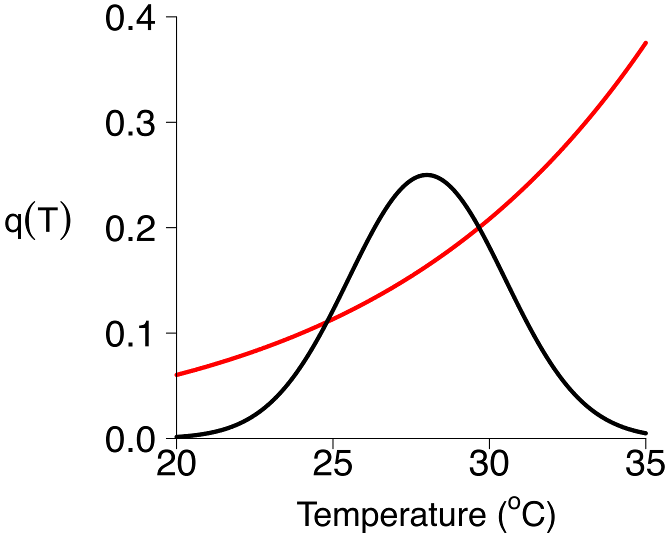

Figure S1. Monotonic increasing (red curve; Eq. (6) in main text) and unimodal (black curve; Eq. (7)) functions depicting how self-limitation strength (q(T)) changes with temperature. Parameters: $q_{T_R} = 0.1$, $A_q = 11{,}000$, $q_{T_{opt}} = 0.25$, $T_{opt_q} = 301$, and $s_q = 2.5$.

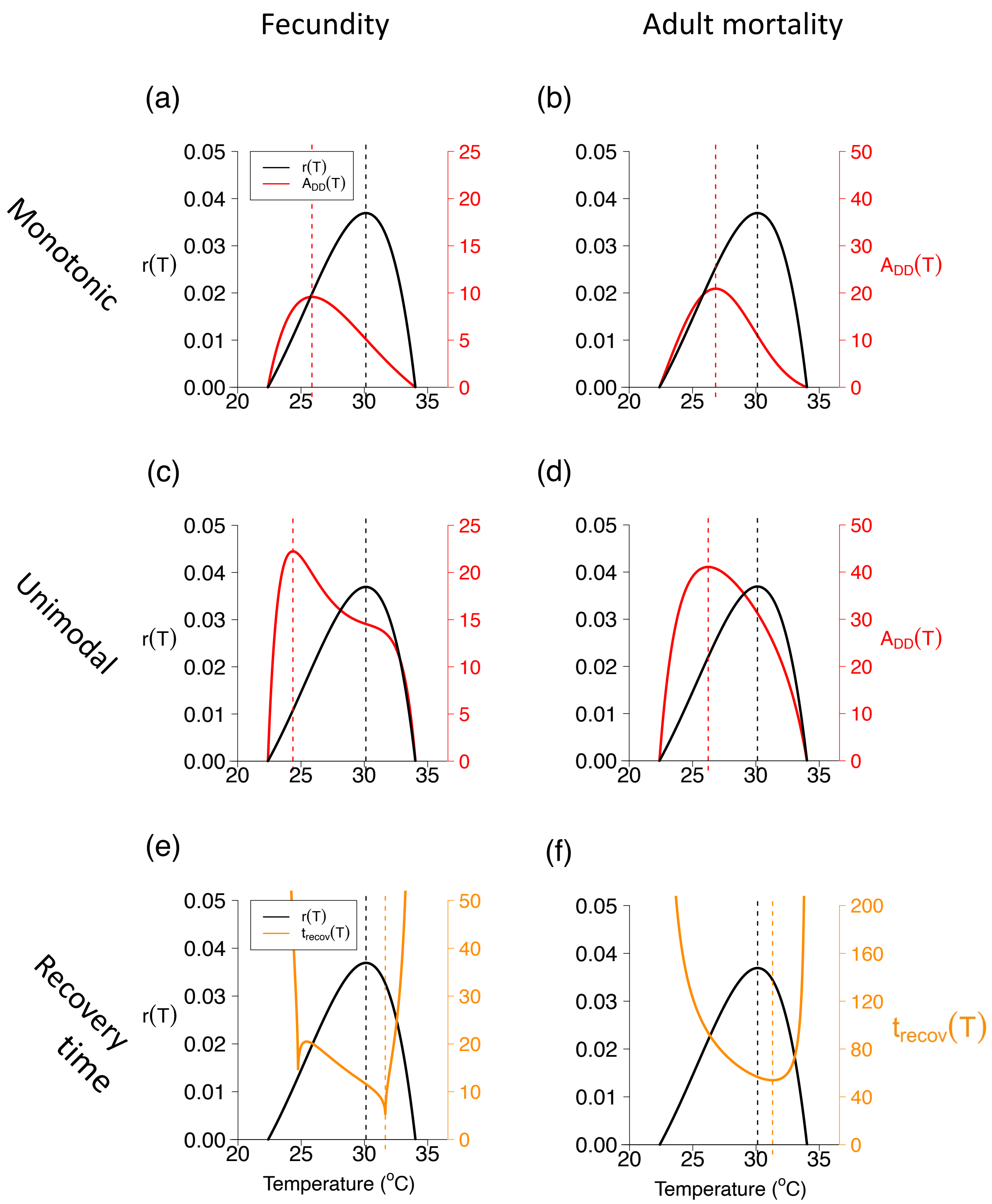

Figure S2. Temperature effects on steady-state adult abundance ($A_{DD}(T)$; red curves), low abundance growth rates ($r(T)$; black curves), and recovery time ($t_{recov}(T)$; orange curves) when density-dependence acts on fecundity (left column) and mortality (right column). Dashed vertical lines indicate the temperature for which each curve achieves its maximum value (or minimum value in the case of recovery time). The top row depicts abundance for a temperature response of competition that is monotonic increasing (panels (a) and (b)), while the middle row depicts abundance for a unimodal response (panels (c) and (d)). The bottom row depicts the

recovery time, which is not affected by the strength of competition (panels (e) and (f); notice that $q(T)$ does not appear in eigenvalue Eqs. (S3) and (S5) above). The eigenvalues associated with temperatures bounded by the two discontinuities of recovery time in panel (e) have real and imaginary parts. Hence, recovery time in that region is slower than immediately outside of the region due to oscillations that occur as the system returns to equilibrium following a perturbation. There are no imaginary parts in eigenvalues associated with the recovery time shown in panel (f). Parameters used are realistic for a warm-adapted species: $b_{T_{opt}} = 2.2$, $T_{opt_b} = 302$, $s = 3.4$, $T_R = 297$, $d_{J_{T_R}} = 0.03$, $A_{d_J} = 6{,}260$, $d_{A_{T_R}} = 0.048$, $A_{d_A} = 14{,}600$, $q_{T_R} = q_{T_{opt}} = 0.1$, $A_q = A_{d_A}$, $T_{opt_q} = T_{opt_b}$, $s_q = s$, $m_{T_R} = 0.015$ and $A_m = 11{,}500$.
