# Supplementary material for: Predicting the fundamental thermal niche of ectotherms
Source: Ecology. Author manuscript; Available in PMC 2025 May 1. (PMC11374413; doi:10.1002/ecy.4289)
Supplement: Appendix S2 [file NIHMS1976892-supplement-Appendix_S2.pdf]

Margaret W. Simon, Priyanga Amarasekare
Predicting the fundamental thermal niche of ectotherms
Supplementary Materials for Article in *Ecology*

This file includes:

- Appendix S2: Main text
- Appendix S2: References
- Appendix S2: Table S1
- Appendix S2: Figure S1

Appendix S2. Comparison of model outcomes when the bagrada's maturation response is bounded versus left unbounded at high temperatures.
Theory and data (Sharpe and DeMichele 1977, Schoolfield et al. 1981, van der Have and de Jong 1996, van der Have 2002, Kingsolver 2009, Kingsolver et al. 2011) show that the temperature response of development rate declines at high temperatures. However, we did not have enough data to fit this declining part of the bagrada bug's maturation curve (right side of red solid curve in Fig. S1 below). We therefore forced the curve to decline at high temperatures using biologically realistic parameters (A_H, $T_{H/2}$; given in Table 1 of the main text) and a maturation response of the form:

$$m_{alt}(T) = \frac{\frac{T}{T_R}\, m_{T_R}\; e^{A_m\left(\frac{1}{T_R} - \frac{1}{T}\right)}}{1 + e^{A_H\left(\frac{1}{T_{H/2}} - \frac{1}{T}\right)}} \qquad (S1)$$

(given in Table 1 notes of the main text).

Alternatively, leaving the function unbounded at high temperatures can be accomplished by fitting a simple exponential maturation function to the data:

$$m_{exp}(T) = m_{T_R}\, e^{A_m\left(\frac{1}{T_R} - \frac{1}{T}\right)} \qquad (S2)$$

(this is the same function as Eq. (13) of the main text; black dashed curve in Fig. S1 below).

Fitting Eq. (S2) to the bagrada development data using the nls() function in R (R Core Team, 2017) gives $A_m = 10{,}944.2 \pm 723.9$ ($p = 6.28 \times 10^{-4}$;). Rerunning our analyses using this parameter value and Eq. (S2) instead of Eq. (S1) has minimal impact on the quantitative results. For example, with Eq. (S2) our niche overlap range does not change from that presented in the main text. Other differences are shown in Table S1 and were calculated in the Appendix S2: Table S1 section of Simulation_FigureGeneration_Rcode.R.

References

Kingsolver JG. 2009. The well-temperatured biologist. *Am Nat* 174: 755–768.

Kingsolver JG, Woods A, Buckley LB, et al. 2011. Complex life cycles and the responses of insects to climate change. *Integrative and Comparative Biology* 51: 719–732.

R Core Team. 2017. R: A Language and Environment for Statistical Computing. R Foundation for Statistical Computing, Vienna, Austria.

Schoolfield RM, Sharpe JH, and Magnuson CE. 1981. Non-linear regression of biological temperature-dependent rate models based on absolute reaction-rate theory. *J Theor Biol* 88: 719–731.

Sharpe PJH and DeMichele DW. 1977. Reaction kinetics of poikilotherm development. *J Theor Biol* 64: 649–670.

van der Have TM. 2002. A proximate model for thermal tolerance in ectotherms. *Oikos* 98: 141–155.

van der Have TM and de Jong G. 1996. Adult size in ectotherms: temperature effects on growth and differentiation. *J Theor Biol* 183: 329–340.

Table S1. Comparison of the bagrada bug's intrinsic growth rate, long-term abundance and recovery time for two different assumptions for the temperature response of maturation

Metric	Form of $m_J(T)$	
	Eq. (S1)	Eq. (S2)
Intrinsic growth rate $r(T)$		
T_{opt_r} (°C)	34.1	34.3
$r_{T_{opt}}$ (per day)	0.058	0.059
T_{min} (°C)	26.8	26.8
T_{max} (°C)	37.7	38.4
Density-dependent fecundity		
$T_{opt_{A_{DD}}}$ (°C)	28.9	28.9
$A_{DD}(T_{opt})$ (adult individuals)	18.1	18.1
$T_{opt_{t_{recovery}}}$ (°C)	35.2	35.3
$t_{recovery_{T_{opt}}}$ (days)	0.451	0.450
Density-dependent mortality		
$T_{opt_{A_{DD}}}$ (°C)	31.3	31.3
$A_{DD}(T_{opt})$ (adult individuals)	8.98	8.96
$T_{opt_{t_{recovery}}}$ (°C)	35.0	35.5
$t_{recovery_{T_{opt}}}$ (days)	0.029	0.031

Figures

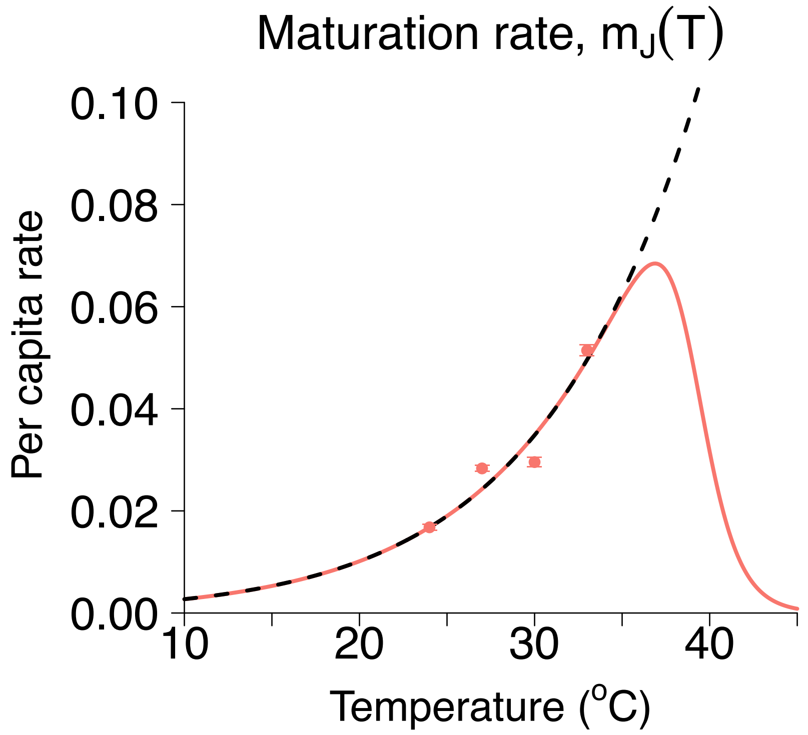

Figure S1. Temperature response of maturation for the bagrada bug. The unimodal function (red solid curve; Eq. (S1)) depicts the full response given in Fig. 1C of the main text (see Table 1 of the main text for parameters). The monotonically increasing function (black dashed curve) is given by Eq. (S2) with $A_m = 10{,}944.2$.
